# Sex Differences in Severity, Social Functioning, Adherence to Treatment, and Cognition of Adolescents with Schizophrenia

**DOI:** 10.1155/2016/1928747

**Published:** 2016-09-14

**Authors:** Rodolfo Pérez-Garza, Gamaliel Victoria-Figueroa, Rosa Elena Ulloa-Flores

**Affiliations:** ^1^Hospital Psiquiátrico Infantil, Mexico City, Mexico; ^2^Arete Proyectos, Mexico City, Mexico

## Abstract

*Background.* Previous studies have reported sex differences in the clinical presentation and outcome of adult patients with schizophrenia; the aim of present study was to compare the clinical characteristics, social functioning, adherence to treatment, and cognition of adolescents with this diagnosis in a six-month followup.* Methods.* A total of 87 adolescents with a DSM-IV diagnosis of schizophrenia or schizophreniform disorder were evaluated with the Positive and Negative Symptoms Scale (PANSS), the Matrics Consensus Cognitive Battery (MCCB), Personal and Social Performance Scale (PSP), and the Rating of Medication Influences (ROMI).* Results.* Both groups showed a similar improvement in all PANSS factors and in the PSP scores during the followup. Males better adhered to treatment. Females displayed better results in the area of social cognition (*F* = 6.3, df = 2,52, and *p* = 0.003) and attention/vigilance (*F* = 8.3, df = 2,51, and *p* = 0.001).* Conclusions.* Male and female adolescents showed similar clinical presentation and functioning but a different pattern of cognitive improvement and adherence to treatment. This trial is registered with Clinicaltrials.gov II3/02/0811.‏

## 1. Introduction

Few studies had examined gender differences in patients with schizophrenia. Highlights from these studies showed that males had an earlier onset [1], more negative symptoms, and a poorer functioning [2], while females showed a higher prevalence of paranoid subtype and more severe positive symptoms and a better outcome [3].

Regarding cognitive function, males were reported to exhibit more difficulties in emotion perception, verbal memory, and cognitive flexibility [4, 5]. A more recent study on patients with first episode of psychosis reported that women obtained higher scores than men on a test of verbal memory, whereas men scored higher on a test of reaction time, visual memory, and planning, displaying a pattern similar to that reported on healthy individuals [6]. Follow-up studies showed similar rates of adherence to treatment [7] but a better outcome in females [8].

Adolescent onset schizophrenia is relatively common, with almost 30% of patients having an illness onset before they turn 18 years old. The male-female ratio for adolescent onset schizophrenia has been reported to be in the range of 1.4 : 1 to 2.2 : 1 in early epidemiological studies [9]. In adolescence, factors such as the increase of hormonal levels and the maturation of the structures and functions involved in the information processing could account for sex differences [10]. For example, a greater loss of cerebral gray matter during brain maturation in males or the protective role of the estrogens in females [11] may in turn affect fundamental aspects of the illness. Given the paucity of studies on this matter, we aimed to compare the clinical characteristics, social functioning, and cognition of male and female adolescents with schizophrenia and to analyze possible differences in early course and adherence to treatment during a six-month followup.

## 2. Methods

Male and female subjects, aged 12 to 17 years, were recruited from the Child Psychiatric Hospital in Mexico City. All met DSM-IV criteria for schizophrenia or schizophreniform disorder [12] based on a diagnostic semistructured interview. Exclusion criteria were active medical comorbidities, drug abuse, and pregnancy. The study was in compliance with the Helsinki declaration and was approved by the Institutional Review Board.

### 2.1. Measures

#### 2.1.1. Diagnosis Was Confirmed with the Mini-International Neuropsychiatric Interview

Child and Adolescent Version (MINI KID): the MINI KID was designed as a structured diagnostic interview to assess short-term psychopathology of children and adolescents. It examines the presence of 23 psychiatric disorders at the present time and throughout life according to DSM-IV and ICD-10 criteria [13].

#### 2.1.2. The Positive and Negative Syndrome Scale (PANSS)

This scale evaluates the severity of symptoms through 30 items which are scored on 1 to 7 scale. A Spanish validated version was used [14] and the severity of symptoms was evaluated according to the five-factor dimensional model of schizophrenic symptoms (positive, negative, excitement, anxiety/depression, and cognitive) [15, 16].

#### 2.1.3. The Personal and Social Performance Scale (PSP)

This is a short instrument with a score of 1–100 points. Lowest values represent a lack of autonomy in the basic functioning, while the highest values reflect excellent performance. Scores are based on an evaluation of the four domains rated according to specific operational definitions: (a) socially useful activities, including work and study, (b) personal and social relationships, (c) self-care, and (d) disturbing and aggressive behaviors [17]. In Mexico, PSP validity was evaluated in 40 adolescent patients, obtaining good internal consistency, a positive correlation with C-GAS, and a negative correlation with the negative, excitement, and cognitive factors derived from PANSS and MCCB domains; it also showed good internal consistency and interrater reliability [18].

The adherence to treatment was defined in a dichotomous variable, assessing the patient's capability to follow the medical instructions [19].

#### 2.1.4. Rating of Medication Influences (ROMI)

This scale was created for the assessment of perceived influences on compliance with antipsychotic treatment. It is composed of 3 Likert-type subscales related to compliance (prevention, influence of others, and medication affinity) and 5 subscales related to noncompliance (denial/dysphoria, logistical problems, rejection of label, family influence, and negative therapeutic alliance) [20].

#### 2.1.5. The Matrics Consensus Cognitive Battery (MCCB)

This instrument is used to evaluate 7 cognitive domains affected by this disorder: speed of processing, attention-vigilance, working memory, verbal learning, visual learning, reasoning/problem solving, and social cognition. The seventh domain, social cognition, was included given its promising nature as a mediator of neurocognitive effects on functional outcome [21]. MCCB performance has been examined in samples of healthy adolescents to determine age adapted standardization [22] and compare the cognitive abilities of psychotic adolescents with those of healthy controls [23, 24]. The reliability was established before the study was developed on every scale using case vignettes and videotapes. Intraclass correlation coefficients > 0.70 and 80% of agreement were achieved.

### 2.2. Procedures

Evaluations were completed at baseline and every three weeks until week 12. PANSS and adherence to treatment were examined on each visit. ROMI was rated on week 3, month 3, and month 6. Other scales were rated on month 3 and month 6. All participants were on pharmacological treatment during the study.

### 2.3. Statistical Analysis

Demographic and clinical characteristics were examined using descriptive statistics. Univariate analysis included chi-square and Student's *t*-tests. The changes in PANSS and MCCB scores were assessed using repeated measures ANOVA adjusted for years of education. If a significant effect was detected, Bonferroni corrections for multiple comparisons were performed to examine the effect of gender, the effect of time, or the differences in change over time among the gender groups (interaction effect). Finally, the Pearson correlation coefficient was used to find the correlations between ROMI's items and adherence to treatment. Significance was set at *p* < 0.05 for all tests. Analyses were run with SPSS 20.0 for Windows.

## 3. Results

### 3.1. General Description of the Sample

The sample included 87 Hispanic adolescents (69% males), with a mean age of 14.9 (±1.5) years, and all were single. Most of them (84%) were on their first episode of psychosis, their mean PANSS score was 98.6 (±21.5), and their mean global PSP was 35.7 (±13.6). Men had a longer mean duration of illness and a lower frequency of first psychotic episode (Table 1).

### 3.2. Psychotic Symptoms and Functioning

Risperidone was the most frequent antipsychotic prescribed (86.7% of males, 70.4% of females, *X* ^2^ = 3.29, df = 1, and *p* = ns). There was a significant improvement in all PANSS factors during the followup (Table 2).

### 3.3. Cognitive Functioning

Both groups showed an improvement on cognitive functioning. Females showed better results in social cognition and attention/vigilance (Table 3).

### 3.4. Social Functioning

Both groups exhibited a poor social functioning on the baseline evaluation. In particular, a larger percentage of the male sample exhibited disturbing/aggressive behavior. The PSP subscales showed a significant improvement during the followup (Table 4). At the sixth month of evaluation the mean PSP global score was 60.5 ± 17 in males and 67.4 ± 11.8 in females.

### 3.5. Adherence to Treatment

**Figure 1:**
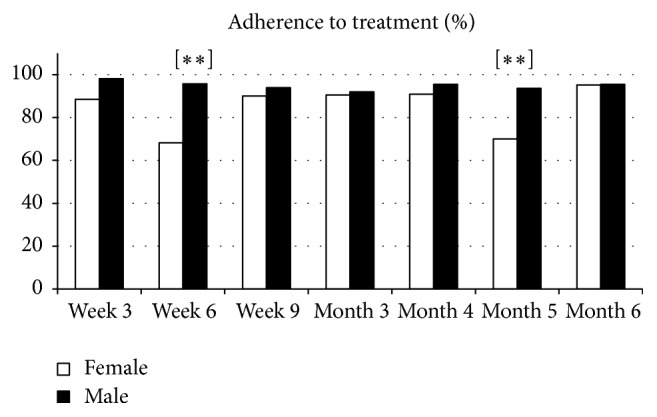
Comparative changes in percentage of adherence to treatment. ^*∗∗*^ *p* < 0.01.

Male adherence was above 95% during the followup. Females showed an inconsistent adherence to treatment and never outnumbered the male subjects. There were significant differences in week 6 (*N* = 45, 95.7% versus *N* = 15, 68.2%, *X* ^2^ = 10.03, df = 1, and *p* = 0.002) and month 5 (*N* = 44, 93.6% versus *N* = 14, 70%, *X* ^2^ = 6.73, df = 1, and *p* = 0.009) (Figure 1). Adherence correlated with specific ROMI items in males (Table 5), while only a negative correlation was found between pressure/force to take medication and adherence to treatment in females (*r* = −0.55, *p* = 0.009).

## 4. Discussion

This study examined sex differences in clinical characteristics and treatment response of adolescents with schizophrenia, an age group on which there is a paucity of studies. The main results included a higher adherence to treatment in males and sex differences in the pattern of cognitive recovery.

The baseline assessment showed an earlier onset of illness in males, which is consistent with previous reports [1, 25, 26]. In contrast with adult studies [2], men showed a higher baseline score in the depression/anxiety factor and higher scores in the excitement factor. Both groups had a significant clinical improvement throughout the followup. Although nonstatistically significant, women showed a higher reduction in the negative factor, which could be related to the males' more severe negative symptoms reported in other follow-up studies [8, 27].

In the current study, female subjects showed a better performance in social cognition and attention/vigilance domains. Previous studies examining sex differences in cognitive development have shown contrasting results, mainly due to the use of different tests [28, 29]. Studies also examining cognitive performance as measured by the MCCB in healthy adolescents reported sex differences in the reasoning and problem solving domain [22, 30]. To the best of our knowledge, this is the first study examining gender differences in adolescents with schizophrenia using the MCCB. The better performance of females in social cognition resembles that of adults, supporting the notion that cognitive development shows a strong improvement during childhood, a moderate improvement in adolescence, and only a slight improvement in late adolescence and young adulthood [31]. This could be in line with previous reports of a better performance in emotion recognition [32] and a lower reduction of amygdala and insula, brain structures mediating emotional and empathy processes in females [25]. The observed differences in the attention/vigilance domain could be explained by the poor performance that males exhibited during followup, reflecting the sustained attention deficits reported in patients with an early onset of the illness [33].

The baseline PSP scores showed that most patients were unable to perform socially useful activities and maintain social relationships. The higher percentage of males showing disturbing/aggressive behaviors was also reported in samples of chronic adult outpatients [26]. Interestingly, both groups exhibited an improvement in all areas, in contrast with adult studies, where women reported a better occupational [34] and social functioning [35]. Present results could be explained by the use of PSP which evaluates particular areas of functioning instead of global functioning.

Male adherence to treatment was better and correlated with a positive family belief, which is in line with results of a study of the first episode of psychosis in adults showing that males received more help from their families, in particular in terms of health, psychotic symptoms, and psychological distress [36]. Future studies could determine whether the families' expectations vary according to the patient's gender and in turn influence the adherence to treatment.

## 5. Limitations

Present results should take into account the small sample size, the short followup, and the lack of evaluation of the family functioning or rearing practices that could provide more information about the psychosocial variables associated with functioning or adherence to treatment.

## 6. Conclusions

Male and female adolescents showed similar clinical presentation and functioning but a different pattern of cognitive improvement and adherence to treatment. Such factors should be considered in the long-term therapeutic programs for this age group.

## Figures and Tables

**Table 1:** Demographic and clinical characteristics of male and female patients.

	Male (*N* = 60)	Female (*N* = 27)	Statistics
*Demographic characteristics*			
Mean age	15.0 ± 1.46	14.78 ± 1.69	*t* = 0.59, df = 44, *p* = ns
Mean number of years of education	7.98 ± 1.74	8.77 ± 1.36	*t* = 2.09, df = 85, *p* = 0.04

*Psychiatric history*			
Mean duration of illness (years)	1.31 ± 1.42	0.76 ± 0.73	*t* = 1.8, df = 85, *p* = 0.05
Mean age of illness onset	13.69 ± 2.11	14.01 ± 1.63	*t* = 0.77, df = 63, ns
First psychotic episode	78.3% (*n* = 47)	96.3% (*n* = 26)	*X* ^2^ = 4.45 , df = 1, *p* = 0.03

**Table 2:** Repeated measures ANOVA for PANSS factors.

	Males	Females	Time effect	Gender effect	Time *∗* gender effect
	Mean (SD)	Mean (SD)			
*Positive*					
Baseline^*∗*^	18.8 (3.7)	17.6 (2.7)	*F* = 119.95, df = 2,56, *p* < 0.001	*F* = 0.68, df = 1,56, *p* = NS	*F* = 0.02, df = 2,56, *p* = NS
Month 3	9.4 (5.5)	8.6 (5.2)			
Month 6	7.6 (4.2)	6.9 (5.2)			

*Negative*					
Baseline^*∗*^	21 (7.5)	21.7 (4.9)	*F* = 41.35, df = 2,57, *p* < 0.001	*F* = 2.03, df = 1,57, *p* = NS	*F* = 0.76, df = 2,57, *p* = NS
Month 3	15.7 (6.4)	12.4 (4.8)			
Month 6	13.8 (6.1)	11.6 (6.5)			

*Excitement*					
Baseline^*∗*^	16.2 (5.1)	13.7 (4.8)	*F* = 32.5, df = 2,54, *p* < 0.001	*F* = 4.39, df = 1,54, *p* = 0.04	*F* = 0.07, df = 2,54, *p* = NS
Month 3	9.6 (5.2)	7.3 (2.9)			
Month 6	8.4 (4.6)	7.7 (4.1)			

*Depression/anxiety*					
Baseline^*∗*^	19.9 (6.4)	16.1 (5.7)	*F* = 23.8, df = 2,55, *p* < 0.001	*F* = 4.77, df = 1,55, *p* = 0.03	*F* = 1.8, df = 2,55, *p* = NS
Month 3	11.4 (3.7)	11.1 (3.3)			
Month 6	11 (4.4)	11.3 (5.7)			

*Cognitive*					
Baseline^*∗*^	20.6 (6.3)	17.7 (5.8)	*F* = 37.05, df = 2,53, *p* < 0.001	*F* = 3.19, df = 1,53, *p* = NS	*F* = 0.1, df = 2,53, *p* = NS
Month 3	13 (6.1)	10.9 (4.1)			
Month 6	12 (5.1)	10.7 (6.3)			

^*∗*^Significant after Bonferroni correction time analysis.

**Table 3:** Repeated measures ANOVA for MCCB.

	Males	Females	Time effect	Gender effect	Time *∗* gender effect
	Mean (SD)	Mean (SD)			
*Speed of processing*					
Baseline	23.2 (14.2)	21.8 (13.5)	*F* = 1.39, df = 2,55, *p* = NS	*F* = 0.00, df = 1,55, *p* = NS	*F* = 1.00, df = 2,55, *p* = NS
Month 3	29.2 (10.8)	31.5 (12.1)			
Month 6	32.7 (12)	32.8 (16.1)			

*Attention/vigilance*					
Baseline^*∗*^	23.1 (8)	20.2 (9.3)	*F* = 3.73, df = 2,51, *p* = 0.03	*F* = 0.53, df = 1,51, *p* = NS	*F* = 8.32, df = 2,51, *p* = 0.001
Month 3^*∗*^	28.3 (9.7)	30.0 (8.1)			
Month 6^*∗*,a^	30.4 (9.1)	36.8 (7.5)			

*Working memory*					
Baseline	31.2 (10.6)	24.4 (14.3)	*F* = 2.74, df = 2,55, *p* = 0.07	*F* = 4.28, df = 1,55, *p* = 0.04	*F* = 1.22, df = 2,55, *p* = NS
Month 3	35.6 (8.7)	31.7 (11.6)			
Month 6	38.1 (9.8)	35.2 (11.5)			

*Verbal learning*					
Baseline	34.9 (9.7)	35.7 (7.6)	*F* = 0.35, df = 2,55, *p* = NS	*F* = 0.12, df = 1,55, *p* = NS	*F* = 0.10, df = 2,55, *p* = NS
Month 3	38.4 (10.1)	40.1 (8.2)			
Month 6	40.6 (10.3)	42 (6.9)			

*Visual learning *					
Baseline	37.1 (12.2)	32.9 (12.6)	*F* = 0.09, df = 2,55, *p* = NS	*F* = 2.54, df = 1,55, *p* = NS	*F* = 1.43, df = 2,55, *p* = NS
Month 3	42 (12.1)	42.5 (10.7)			
Month 6	43.6 (10.6)	44.6 (10.5)			

*Reasoning and problem solving*					
Baseline^*∗*^	37.1 (5.4)	37.2 (7.7)	*F* = 3.29, df = 2,55, *p* = 0.04	*F* = 1.68, df = 1,55, *p* = NS	*F* = 2.09, df = 2,55, *p* = NS
Month 3^*∗*^	40.1 (6.2)	38.1 (4.5)			
Month 6^*∗*^	44.1 (9.0)	39.9 (5.8)			

*Social cognition*					
Baseline	30.9 (9.9)	28.3 (9.1)	*F* = 0.27, df = 2,52, *p* = NS	*F* = 2.13, df = 1,52, *p* = NS	*F* = 6.33, df = 2,52, *p* = 0.003
Month 3	27.5 (9.6)	30.6 (13.2)			
Month 6^a^	27.4 (9.2)	36.3 (12.9)			

*Overall composite*					
Baseline	20.6 (10.7)	19.6 (10.9)	*F* = 2.44, df = 2,49, *p* = NS	*F* = 0.42, df = 1,49, *p* = NS	*F* = 2.65, df = 2,48, *p* = NS
Month 3	25.7 (10.3)	28.8 (9.8)			
Month 6	29.2 (10.7)	33.7 (8.8)			

^*∗*^Significant after Bonferroni correction time analysis and ^a^significant after Bonferroni correction sex analysis.

**Table 4:** Percentage of subjects showing a good functioning in the PSP subscales during the followup.

	Males	Females	*X* ^2^	df	*p*
	*N* (%)	*N* (%)			
*Baseline*					
(1) Socially useful activities	2 (3.3)	1 (3.7)	0.01	1	0.93
(2) Personal and social relationships	3 (5)	1 (3.7)	0.07	1	0.79
(3) Self-care	14 (23.3)	7 (25.9)	0.07	1	0.79
(4) Disturbing and aggressive behaviors	24 (40)	20 (74.1)	8.65	1	0.01

*Month 3*					
(1) Socially useful activities	18 (36)	9 (40.9)	0.16	1	0.69
(2) Personal and social relationships	17 (34)	7 (31.8)	0.03	1	0.86
(3) Self-care	37 (74)	17 (77.3)	0.09	1	0.77
(4) Disturbing and aggressive behaviors	42 (84)	19 (86.2)	0.07	1	0.80

*Month 6*					
(1) Socially useful activities	18 (41.9)	13 (61.9)	2.27	1	0.13
(2) Personal and social relationships	14 (32.6)	12 (57.1)	3.54	1	0.06
(3) Self-care	31 (72.1)	18 (87.5)	1.46	1	0.23
(4) Disturbing and aggressive behaviors	38 (88.4)	19 (90.5)	0.06	1	0.8

**Table 5:** Correlation between the adherence to treatment and ROMI items in males.

	Week 3	Month 3	Month 6
*Males*			
(1) Perceived benefit	—	−0.3^*∗*^	—
(2) Positive relationship with clinician	—	−0.33^*∗*^	—
(3) Positive relationship with therapist	—	—	—
(4) Positive family belief	0.28^*∗*^	0.36^*∗*^	—
(5) Relapse prevention	—	—	0.31^*∗*^
(6) Pressure/force	−0.3^*∗*^	—	—
(7) Fear of rehospitalization	—	−0.39^*∗∗*^	—

^*∗*^ *p* ≥ 0.05; ^*∗∗*^ *p* ≥ 0.01.
